# Defect Recognition in Composite Materials Using Terahertz Spectral Imaging with ResNet18-SVM Approach

**DOI:** 10.3390/ma18112444

**Published:** 2025-05-23

**Authors:** Zhongmin Wang, Jiaojie Chen, Yilong Xin, Yongbin Guo, Yizhang Li, Huanyu Sun, Xiuwei Yang

**Affiliations:** 1 Institute of Automation, Qilu University of Technology (Shandong Academy of Sciences), Jinan 250100, China; 2 School of Information Science and Engineering (School of Software), Yanshan University, Qinhuangdao 066000, China

**Keywords:** terahertz wave, ResNet18, SVM, deep learning, nondestructive testing

## Abstract

Multilayer composite materials often develop internal defects at varying depths due to manufacturing and environmental factors. Traditional planar scanning methods lack the ability to pinpoint defect locations in depth. This study proposes a terahertz time-domain spectroscopy (THz-TDS)-based defect detection method using continuous wavelet transform (CWT) to convert spectral signals into time-frequency images. These are analyzed by the ResNet18 model combined with a support vector machine (SVM) classifier. Comparative experiments with four classical deep learning models and three classifiers show that the Residual Network with 18 layers (ResNet18-SVM) approach achieves the highest accuracy of 98.56%, effectively identifying three types of defects. The results demonstrate the method’s strong feature extraction, depth resolution, and its potential for nondestructive evaluation of multilayer structures.

## 1. Introduction

The composite sandwich structure is the most prevalent type of multilayer structure, offering advantages in cost, weight, fatigue resistance, specific stiffness, and specific strength. Consequently, they are widely employed in several industries, including aerospace, wind turbine blades, automotive, and shipbuilding [1]. Representative products include radomes, wings, and engine sound insulation panels of airplanes [2,3]. The composite sandwich structure comprises a lightweight, thicker core and a thinner fiberglass composite skin [4,5,6]. However, during the preparation and use of composite sandwich structures, some defects are inevitable due to non-ideal production environment, human error, and other factors, which strongly impact the overall mechanical performance and should be detected promptly [7]. Defects in composite sandwich structures vary in depth and challenge the traditional inspection methods regarding two-dimensional imaging. However, as the demand for composite sandwich structures in all fields grows dramatically, the quality criterion becomes increasingly strict. Furthermore, the industry’s meticulous classification and identification of defects have become urgent [8].

Terahertz spectroscopic imaging allows non-contact, non-ionizing, and nondestructive tests of various non-metallic materials with multilayer structures [9,10,11]. In the nondestructive testing of composite sandwich structures, continuous scanning is achieved to obtain the terahertz time-domain spectral signals for each point on its surface [12]. Then, terahertz images of the composite core structure are generated via various imaging techniques [13]. By processing terahertz images, investigators analyze and obtain the number of defects and the morphology [14]. Furthermore, the terahertz time-domain signals contain information on the amplitude and phase of the transient electric field, which can be transformed into the frequency domain using Fourier analysis. This enables accurate characterization of multilayer structures, particularly in terms of defect depth [15,16,17]. Consequently, integrating spectral information and image data enables a comprehensive understanding of the number, position, and morphology of defects within the multilayer structure of composite materials [18].

The quality of terahertz images is significantly affected by the limited signal-to-noise ratio. When experimenters rely on the original images to manually identify defects, the conclusions are often unconvincing and unreliable [17,19,20]. In addition, traditional methods require manual predefinition of spectral features and are prone to human interference, which negatively impacts classification accuracy. In contrast, deep learning algorithms greatly improve the detection accuracy and generalization capability for delamination defects in quartz fiber-reinforced polymer (QFRP) materials through automated feature extraction and efficient classification. When combined with terahertz time-domain spectroscopy (THz-TDS), a Transformer-based neural network model achieves an impressive F1-score of 0.982, enabling reliable defect visualization while effectively preventing false identification in defect-free regions [21]. Furthermore, studies have demonstrated that deep learning algorithms, when integrated with terahertz nondestructive evaluation techniques such as THz-TDS, can significantly enhance the detection and localization of internal cable defects by leveraging intelligent classification and automated feature extraction. These methods enable high-precision measurement of cable sheath thickness and overcome the limitations of conventional nondestructive testing techniques in defect characterization [22].

To address the challenge of accurately identifying internal defects in multilayer composite sandwich structures, this study presents an integrated method combining THz-TDS, continuous wavelet transform (CWT), and a deep learning classification framework. Specifically, CWT is applied to convert time-domain terahertz signals into time-frequency images, which are then input into a Residual Network with 18 layers (ResNet18) to extract deep features. To improve classification robustness, particularly under limited data conditions, the extracted features are further classified using a support vector machine (SVM). This joint ResNet18-SVM approach effectively leverages the representational power of deep networks and the generalization ability of SVM. Experimental results show that the proposed method achieves the highest classification accuracy of 98.56%, demonstrating its strong potential for intelligent, non-contact, and nondestructive defect detection in aerospace-grade composite structures.

## 2. Experimental Design

### 2.1. Structural Design

**Figure 1:**
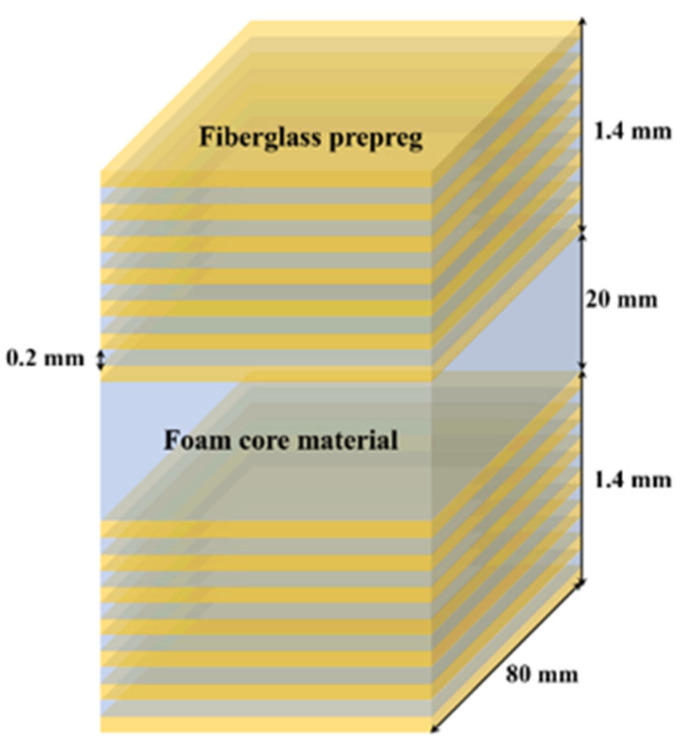
Design of a sandwich structure of composite multilayer structure.

Our study employs a composite multilayer structure to simulate aircraft radomes [23]. The structure comprises an upper skin, a lower skin, an adhesive, and an intermediate core. Both the upper and lower skins are made of glass fiber-reinforced composite laminates with excellent wave-transmitting properties. Each skin has a total thickness of 1.4 mm, consisting of seven prepreg layers arranged at lay-up angles of 0°/90°/45°/−45°. To simulate delamination defects commonly observed in multilayer composite structures under real-world conditions, a 0.2 mm-thick polytetrafluoroethylene (PTFE) film was intentionally inserted between the third and fourth prepreg layers of the fiberglass laminate. This design effectively mimics multilayer composite sandwich structures commonly used in aerospace applications such as radomes and wing panels. The adhesive provides a strong interfacial bond between the skins and the core. These skins are bonded to a lightweight PMI foam core using a structural adhesive, forming a typical sandwich configuration. Polymethacrylimide (PMI) foam, a material widely used in aerospace structures, is employed as the intermediate core, with the FOAM-MH model selected for this study [24]. Figure 1 illustrates a schematic diagram of this composite sandwich structure.

### 2.2. Manual Defect Preset

**Figure 2:**
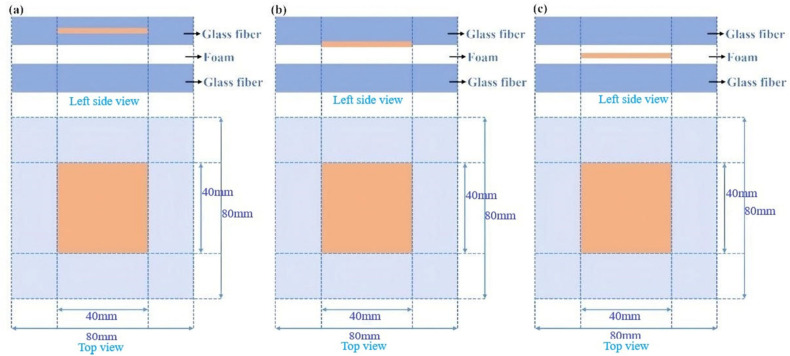
Top view of different types of defects: (**a**) delamination defect, (**b**) adhesion failure defect, and (**c**) void defect.

There are three primary defects in composite sandwich structures: adhesion failure, layer delamination, and void defect. These defects are distributed at different depths randomly in the actual state. PTFE was used in the experiment to simulate the air gap commonly found in delaminated regions. This artificial interface mimics the air gap typically observed in delaminated regions. PTFE was selected due to its low refractive index (approximately *n* ≈ 1.43), which is close to that of air (*n* ≈ 1.0), thereby creating a similar electromagnetic contrast during terahertz wave propagation. Therefore, PTFE is commonly used to simulate delamination defects [25,26]. To evaluate the ability of the ResNet18 network to detect these defects, the shape, size, and depth of the defects to be detected were designed in this study. Figure 2 illustrates the top view and left side view of defects whose detailed information is found below:

1. Layer delamination: To simulate delamination defects commonly found in multilayer composite structures under real-world conditions, a 0.2 mm thick PTFE film was deliberately inserted between the third and fourth prepreg layers of the fiberglass composite as shown in Figure 2a.
2. Adhesion failure: In this experiment, to simulate debonding defects in fiberglass materials bonded with foam adhesive, a 0.2 mm thick PTFE sheet was placed in a non-adhesive state, as shown in Figure 2b.
3. Void defect: Cavities in different sizes, shapes, and depths were created on the surface of the foam material and are used to simulate cavity defects, as shown in Figure 2c.

**Figure 3:**
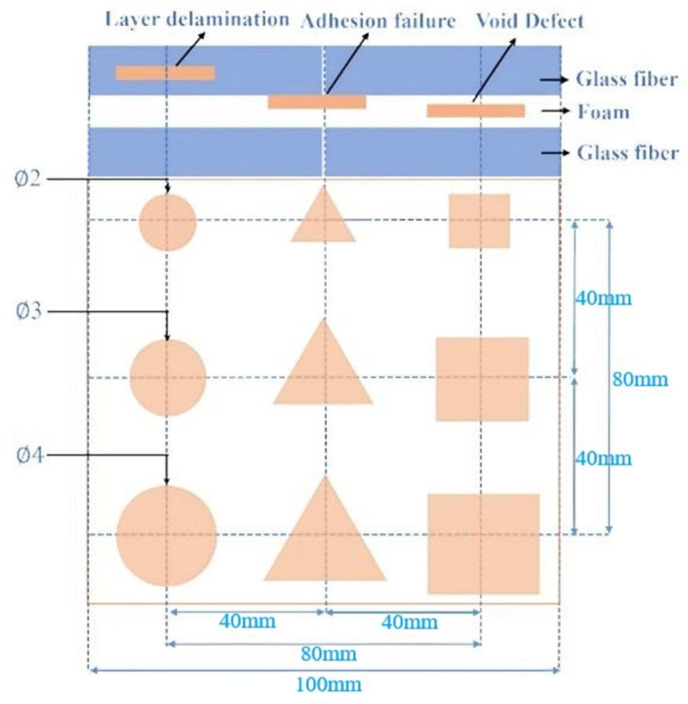
Distribution map of three types of defects.

To verify the accuracy of the proposed algorithm for classifying and identifying the three defects, PTFE sheets were embedded inside the samples to simulate these defects. Delamination defects were simulated using PTFE circular sheets with a thickness of 0.2 mm and diameters of 4 mm, 3 mm, and 2 mm, respectively. PTFE triangular sheets were used to simulate adhesion failure defects, representing a non-adhesive state. The side lengths are 4 mm, 3 mm, and 2 mm, respectively, with a thickness of 0.2 mm. Void defects are created in the foam using grooves, and their shape is a square with a uniform depth of 2 mm but with varying side lengths. The side lengths are 4 mm, 3 mm, and 2 mm, respectively. The dimension of the prepared defect is shown in Figure 3.

**Figure 4:**
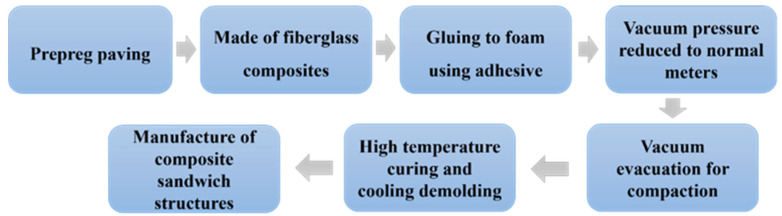
Flowchart for the preparation of integral sandwich knots.

The preparation process of this experiment was carried out following the requirements of Q/ML-PS1 “Fiber Reinforced Composites Hot Press Canning Process”. The overall preparation process is shown in Figure 4 and is divided into the following steps: The prepreg is first stacked and prepared as a fiberglass composite, then the fiberglass is stuck on the foam using adhesive and compacted by vacuum evacuation. It should be noted that to prevent collapse at the cavity defects during vacuum pressing. Therefore, the vacuum pressure is reduced to half of the normal value. Finally, the sample is cured at high temperature, cooled, and molded into a composite sandwich structure.

### 2.3. Signal Acquisition

**Figure 5:**
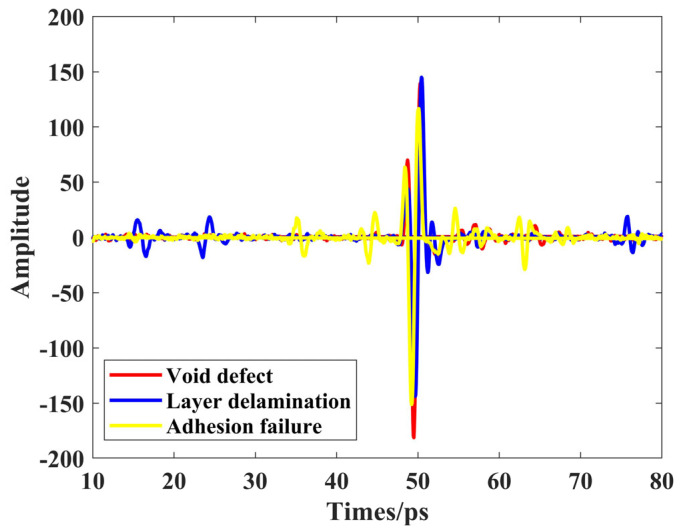
Terahertz time-domain signals of three types of defects. Red line, blue line, and yellow line are layer delamination, void defect, and adhesion failure, respectively.

In this experiment, a THz-TDS (15-FCO-STDX-005, Zomega, New York, NY, USA) system in reflection mode was used to scan the samples with a step size of 2 mm. The THz spot size on the sample surface is approximately 0.5 mm in diameter. As shown in Figure 5, the terahertz time-domain signals collected from the locations of the three types of defects indicate that layer delamination located on the upper layer has signal echoes from their top and bottom surfaces appearing between 10–30 ps. The adhesion failure, situated in the middle, has its top and bottom surface echoes appearing between 30–50 ps. Lastly, void defects are located on the lower layer, with their surface signal echoes appearing between 50–70 ps. This time delay corresponds directly to the depth of the defects within the material, as the terahertz waves travel at a relatively constant speed through the medium. Therefore, echoes appearing at later times indicate reflections from deeper layers, enabling the precise localization of defects by analyzing the temporal position of the echoes.

### 2.4. Terahertz Spectral Signal Feature Extraction

The time-frequency diagram converts a one-dimensional signal into an image that reflects the variation of frequency over time. Commonly used time-frequency analysis methods include short-time Fourier transform (STFT), empirical mode decomposition (EMD), and wavelet transform. Among them, the CWT offers better time-frequency localization than STFT due to its multi-resolution characteristics. Compared with EMD, which often suffers from mode mixing and lacks a solid theoretical foundation, CWT produces more stable and interpretable results. By adaptively adjusting time-frequency resolution, the wavelet transform demonstrates superior performance among various methods [27,28,29]. Moreover, it can simultaneously present time, frequency, and energy information in a single diagram. Therefore, we chose the wavelet transform to generate time-frequency diagrams of the three types of defect signals, which serve as inputs to the convolutional neural network for feature extraction and identification.

Wavelet transform has been demonstrated in multiple studies to be effective for THz-TDS analysis. Compared with the Fourier transform, CWT has significant advantages in obtaining both time and frequency information simultaneously, making it especially suitable for analyzing non-stationary signals. CWT not only captures the frequency information of the signal but also indicates when specific frequency components occur in the time domain, making it ideal for analyzing terahertz time-domain signals. For terahertz time-domain spectroscopy signals, CWT is performed by convolving the time-domain signal f(t) with a wavelet basis function, yielding the CWT coefficients:

(1) $$C\left(a,b\right)=\int_{-\infty }^{\infty }f\left(t\right)\frac{1}{\sqrt{a}}\varphi^{*}\left(\frac{t-b}{a}\right)dt$$

where φ* represents the complex conjugate of the wavelet basis function, a is the scale parameter (analogous to frequency), and b is the position parameter (analogous to time delay). By continuously adjusting a and b, matrix C(a, b) of wavelet coefficients is obtained. In this study, the complex Morlet wavelet is chosen as the wavelet basis function, which is expressed as:

(2) $$\varphi \left(t\right)=\frac{1}{\sqrt{\pi f_{b}}}\cdot e^{j2\pi f_{c}t-\left(\frac{t^{2}}{f_{b}}\right)}$$

where f_c_ and f_b_ represent the central frequency and bandwidth of the Morlet wavelet, respectively, both set to 3 THz.

## 3. Convolutional Neural Network Design for Defect Detection in Terahertz Spectral Signal

### 3.1. Resnet18

As a classical deep learning network model, residual networks have demonstrated excellent feature extraction and classification performance. ResNet18, a widely used representative of residual networks, addresses issues such as gradient vanishing and gradient explosion in deep networks by introducing residual modules. This enables the network to train deeper layers while preserving key feature information. ResNet18 effectively fuses shallow and deep features extracted from the CWT time-frequency maps by leveraging residual connections, which is particularly advantageous for multi-scale feature extraction. This enhances sensitivity to critical information within the time-frequency maps. Compared to more complex deep networks, ResNet18 features fewer parameters and greater computational efficiency, making it especially suitable for efficient feature extraction on small and medium-sized datasets. Its ability to avoid overfitting aligns well with the constraints of terahertz datasets, which are often limited in size. Therefore, ResNet18 is an excellent choice for feature extraction in this context, contributing to subsequent classification tasks.

**Figure 6:**
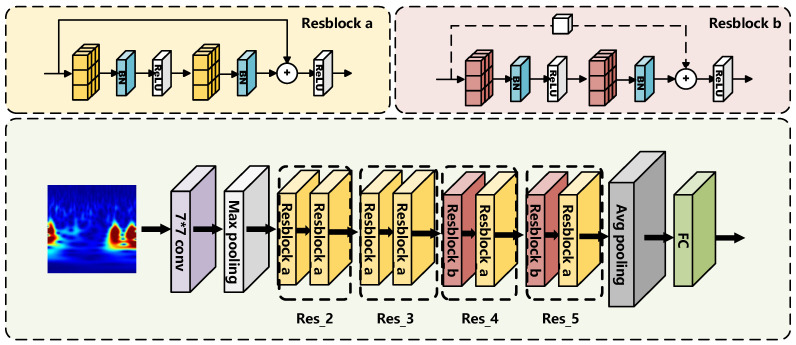
Two Residual blocks and ResNet18 model.

Figure 6 illustrates the two residual block types and the overall ResNet18 architecture. The network inputs time-domain images transformed by CWT. Initial features are extracted using a 7 × 7 convolutional layer followed by max pooling. The network then passes through four residual stages (Res_2 to Res_5), each containing two block types: Resblock a with identity shortcuts maintains input-output dimensions, while Resblock b uses 1 × 1 convolution shortcuts with a stride 2 for downsampling and dimension alignment. This structure enhances multi-scale feature extraction. After the residual stages, global average pooling and a fully connected layer produce the final features for defect classification, effectively mitigating vanishing gradients and improving performance. This step size halves the length and width of the output feature matrix relative to the input. The inclusion of residual blocks in the ResNet18 network enhances the dimensionality of the output features while effectively mitigating issues such as gradient vanishing and network degradation. The size of the convolution kernel in the convolutional layer of the model used is 3 × 3, and the activation function is a Rectified Linear Units (ReLU) function. Among them, the ReLU activation function is a maxima-taking function, the construction of which, although simple, is an essential result in deep learning. The computation and convergence speed are much faster than the Sigmoid and Tanh functions [30]. In ResNet18, the pooling operation is to find a value for a given region on the feature map, as deeper information is employed in the sequential structures. Pooling is essential for reducing computation and preventing overfitting. The feature map will decrease in size after pooling [31]. Max-pooling is used in this model by setting the filter size to 2 × 2 and the step size to 2.

### 3.2. Grid Structure

**Figure 7:**
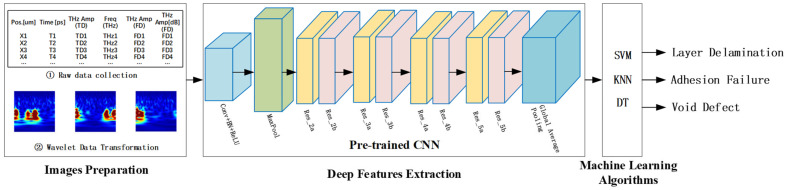
Convolutional neural network for terahertz spectral signal defect detection with its feature extraction method and comparison.

After the ResNet18 model training is completed, feature extraction can be performed on the input image using convolutional neural networks [32]. The learned convolutional filters automatically extract meaningful patterns from the time-frequency images of terahertz spectral signals, such as localized energy distributions and structural features associated with specific defect types. These features, which encode both spatial and frequency domain information, are then flattened into a one-dimensional feature vector. This feature vector is subsequently fed into an SVM classifier to perform the final classification task [33,34]. Given the presence of three defect categories—layer delamination, adhesion failure, and void defect—the binary SVM classifier is extended to a multiclass classification problem using a one-vs-one strategy. The entire classification framework is illustrated in Figure 7. By combining the deep feature extraction capability of ResNet18 with the strong generalization performance of the SVM, this hybrid approach achieves accurate and robust classification of terahertz spectral signal defects. Additionally, the separation of feature extraction and classification processes enhances the interpretability and flexibility of the model.

## 4. Experiment

The experimental platform was built under Windows 10 with an NVIDIA GPU GTX1060 (NVIDIA, Santa Clara, CA, USA), 6G video memory, and 16G RAM configuration, and equipped with MATLAB R2022b environment.

### 4.1. Data Preparation

A rigorous segmentation of the dataset is employed to ensure comprehensive evaluation and performance validation of the model. The original dataset contains 1000 time-frequency 2D plots for each class of defects. Firstly, the original dataset is divided into a training set and a test set, where the training set accounts for 70%, and the test set accounts for the remaining 30%. To optimize the model and avoid overfitting, 30% of the training set is reserved for validation. Four hundred ninety time-frequency maps in each defect type are categorized into the training set, 210 time-frequency maps are categorized into the validation set, and 300 are used for the test set. Such a division makes full use of the information in the dataset and ensures that the training set is used to learn the model parameters. The validation set is used for tuning the hyperparameters, while the test set is used for the final evaluation of the model’s generalization performance. With such appropriate data segmentation, the model’s performance can be assessed in a way that helps reduce overfitting and supports fair model validation, thereby ensuring the credibility of the experimental results.

### 4.2. Model Testing and Analysis

#### 4.2.1. Impact of Different Machine Learning Algorithms on Model Performance

A ResNet-18 model is proposed in this study as the basis for a defect detection method for terahertz spectral signals. The method’s performance is evaluated using four classical deep learning models—AlexNet, Visual Geometry Group 16-layer network (VGG-16), Visual Geometry Group 19-layer network (VGG-19), and ResNet18—combined with three different classification algorithms: SVM, K-Nearest Neighbors (KNN), and Decision Tree (DT). To evaluate these models’ performance in detecting defects in terahertz spectral signals, we employ a dataset containing samples of multiple defect kinds and evaluate it using the common performance metrics, including accuracy, precision, sensitivity, specificity, and AUC.

To evaluate classification performance, standard metrics including precision, recall, and F1-score were computed based on the confusion matrix. These metrics are defined as follows:

(3) $$Precision=\frac{TP}{TP+FP}$$

(4) $$Recall=\frac{TP}{TP+FN}$$

(5) $$F1_{Score}=\frac{2\cdot Precision\cdot Recall}{Precision+Recal}$$

Here, the confusion matrix elements are defined as follows for a given defect category. True Positive (TP): The number of samples correctly predicted as the given defect class; False Positive (FP): The number of samples incorrectly predicted as the given defect class; False Negative (FN): The number of samples belonging to the defect class but incorrectly predicted as another class. F1_Score provides a balanced measure between precision and recall, especially useful in cases of class imbalance. These metrics were computed separately for each defect category (layer delamination, adhesion failure, and void defect) using the corresponding entries in the confusion matrix.

As can be seen in Table 1, the ResNet18 model shows excellent performance under all classification algorithms. In particular, the model achieved an accuracy of 98.56% with the SVM algorithm, which is higher than other models. This demonstrates the superiority and reliability of the ResNet18 model for defect detection in terahertz spectral signals.

It is worth noting that applying the SVM algorithm to AlexNet, VGG-16, and VGG-19 also yielded relatively good results, with accuracies of 96.79%, 95.08%, and 94.96%, respectively, indicating that traditional models can perform well in certain tasks. However, ResNet18 outperformed these models, primarily due to its residual connections, which alleviate the vanishing gradient problem and enable deeper network training. This structure enhances the model’s ability to learn complex features effectively, even with limited data, making ResNet18 particularly suitable for defect detection in terahertz spectral signals.

**Figure 8:**
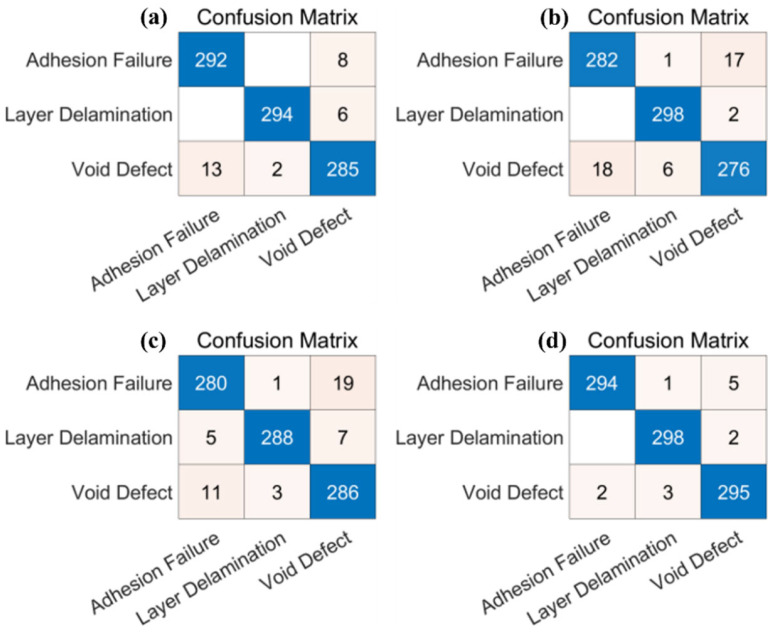
Confusion matrix obtained by (**a**) AlexNet, (**b**) VGG-16, (**c**) VGG-19, and (**d**) ResNet18 combined with SVM algorithm.

To further explore the performance of different deep learning models (AlexNet, VGG-16, VGG-19, and ResNet18) combined with the SVM algorithm in the terahertz spectral signal defect detection task, the classification accuracy of four depth models can be compared through the confusion matrix, as shown in Figure 8. The combination of different models and SVM in the confusion matrix of terahertz spectral signal defect detection shows that the ResNet18 network has higher classification accuracy. In addition, as shown in Table 2, the accuracy rate, recall rate, and F1 score obtained by the four models combined with the SVM algorithm are compared, and the following conclusions can be drawn:

First, it can be observed from the data in Table 2 that ResNet18 combined with SVM achieves the best performance in all categories. Especially for adhesion failure and layer delamination, the precision and recall of the model are close to 1, indicating that the model has excellent accuracy and recall rate in these two categories.

Second, the performance of VGG-16 and VGG-19 pales on some categories when they are combined with SVM. Especially in the Void Defect category, the performance of these two models is mediocre according to precision and recall. It is implied that VGG series models are relatively weak in representing certain categories of features in terahertz spectral signal defect detection tasks.

These results further highlight the advantages of residual structure in the ResNet18 model. By introducing residual learning, ResNet18 effectively solves the problem of gradient vanishing in deep networks, permitting the construction of deeper network structures and improving the model’s performance. Thus, the ResNet18 model combined with SVM excels in defect detection of terahertz spectral signals. In summary, the ResNet18 model combined with SVM achieves the best performance on defect detection, whereas VGG-16 and VGG-19 combined with SVM perform relatively poorly in some categories. This provides a valuable guideline for selecting an appropriate model in terahertz spectral signal defect detection tasks.

#### 4.2.2. Impact of Different Pooling Layers on the Model

**Figure 9:**
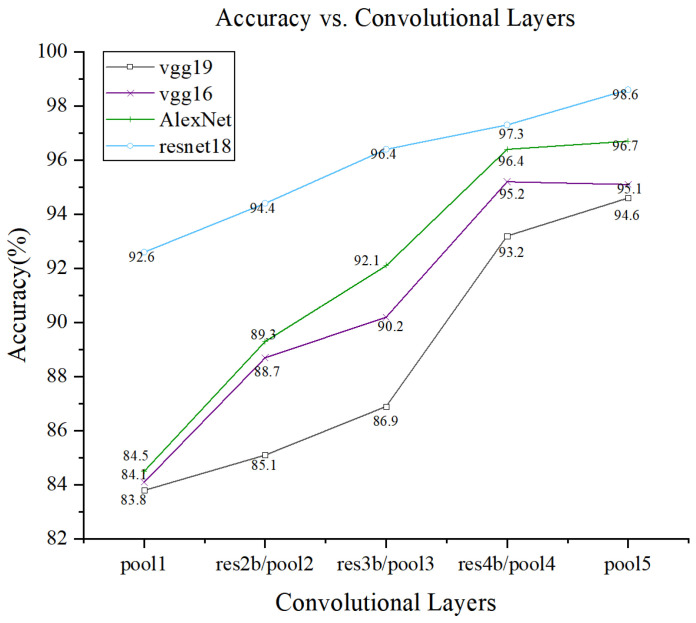
Effect of different model pooling layers on accuracy.

In general, the features extracted from terahertz spectral signal images become increasingly abstract with the depth of the convolutional layers, and the features extracted from different layers have different effects on the classification results. To further visualize the features of the ResNet18 model at different convolutional layers and to gain insight into the extracting process of convolutional neural networks, we compare the features extracted from different layers of the convolutional neural network. As shown in Figure 9, the features in the Pool1, Res2b, Res3b, Res4b, and Pool5 layers of the ResNet18 model are selected to visualize three types of terahertz spectral signals, respectively. The visualizations were generated by adding visualization code after each selected layer to extract and display the corresponding feature maps. Moreover, the features with different depths are used in the classification of the support vector machine algorithm.

To further investigate the impact of different pooling layers on model performance, we analyzed four deep learning models—AlexNet, VGG-16, VGG-19, and ResNet18—by extracting feature representations from various pooling stages (Pool1 to Pool5). For each pooling layer in these models, the corresponding feature maps were used as inputs to an SVM classifier for defect classification. Figure 9 presents the classification accuracy achieved by each model across different pooling layers. The results indicate that deeper pooling layers generally lead to better performance in AlexNet, VGG-16, and VGG-19, likely due to enhanced abstraction of high-level features. In contrast, ResNet18 demonstrates relatively stable performance across all pooling stages, achieving its peak accuracy at the Pool5 layer.

**Figure 10:**
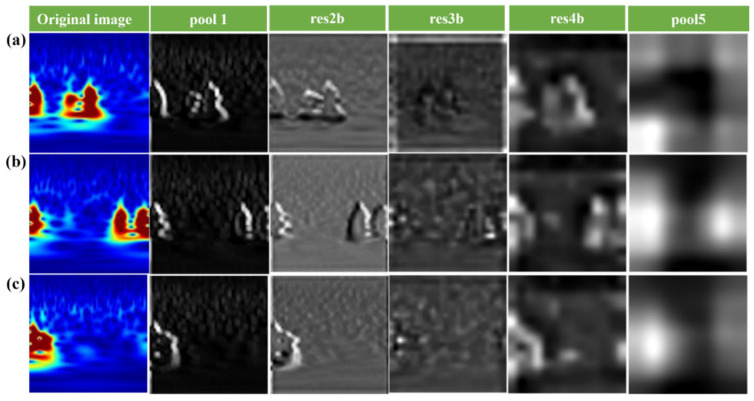
Terahertz spectral signal feature maps of three typical defect types generated by the ResNet18 model and their corresponding feature layer outputs: (**a**) layer delamination, (**b**) adhesion failure, and (**c**) void defect.

To provide a more detailed analysis of pooling layer effects, we further examined the feature extraction capability of each pooling layer by visualizing their corresponding feature maps (Figure 10) and evaluating their classification performance (Figure 9). The results clearly indicate that as the depth of the pooling layer increases, the extracted features become increasingly abstract and more discriminative. For instance, features at Pool1 are relatively low-level, capturing basic edge and texture information, while features at Pool5 represent more semantically meaningful structures relevant to defect types. Moreover, classification accuracy varied significantly across different pooling layers and network architectures. In AlexNet and VGG models, deeper pooling layers (Pool4 and Pool5) yielded higher accuracy, highlighting the importance of deep hierarchical features for defect classification. In contrast, ResNet18 showed relatively stable accuracy across all pooling layers, with Pool5 still achieving the highest performance. This robustness may be attributed to the residual learning mechanism, which preserves important information throughout the network layers.

The results imply that the selection of the appropriate pooling layer is crucial for different deep-learning models on this task. In the AlexNet and VGG models, deeper pooling layers typically outperform shallower ones. On the contrary, pooling layers have relatively little effect on accuracy in ResNet18. These findings provide helpful guidance to optimize model performance and to improve defect detection methods.

Figure 10 presents the visualization of three distinct categories of terahertz spectral signal images, aimed at demonstrating the influence of the pooling layer on the extracted feature representations. By comparing the convolutional feature maps corresponding to these three terahertz spectral classes, it is evident that as the number of convolutional layers increases, the salient characteristics within the terahertz spectral images become progressively more pronounced. This progression highlights the hierarchical nature of feature extraction in deep convolutional neural networks, where initial layers capture basic local patterns, and deeper layers encode increasingly abstract and discriminative features. Consequently, the features extracted from deeper convolutional layers possess higher semantic richness and greater discriminative power, thereby enhancing the model’s ability to accurately classify and detect terahertz spectral signals.

## 5. Conclusions

In conclusion, this study proposes a defect detection method for composite material sandwich structures based on terahertz spectral image analysis using the ResNet18 model. By combining continuous wavelet transform with deep learning and SVM classification, the method achieves high accuracy and robustness in identifying delamination, voids, and adhesion failure defects. The results demonstrate that this approach not only enhances the interpretability of terahertz data but also significantly contributes to the advancement of intelligent, non-contact, and nondestructive inspection tools for ensuring the structural safety of multilayer composite materials, particularly in aerospace and related industries. However, the model was trained on pre-defined defect types and controlled laboratory conditions, which may limit its generalizability to more complex or real-world defect scenarios. Future work should focus on expanding the dataset diversity and validating the model’s performance under practical industrial environments.

1. The ResNet18 model excels in terahertz spectral signal defect detection, especially on condition that SVM is employed sequentially, achieving an accuracy of 98.56%;
2. ResNet18 outperforms the other three deep learning models significantly in terahertz spectral signal defect detection thanks to the residual structure;
3. The impact of pooling layers on model accuracy varies across architectures. While the choice of pooling layer can significantly influence performance in some models, ResNet18 shows stable accuracy regardless of the pooling layer used.

## Figures and Tables

**Table 1:** Performance comparison of different models and algorithms.

Feature Extracting Model	Classification Algorithm	Accuracy (%)	Precision (%)	Sensitivity (%)	Specificity (%)	AUC
AlexNet	SVM	96.7778	96.7933	96.7778	98.3889	0.8901
	KNN	77.6667	81.0744	77.6667	88.8333	0.7591
	DT	70.5556	70.7305	70.5556	85.2778	0.6864
VGG-16	SVM	95.1111	95.0881	95.1111	97.5556	0.8769
	KNN	67.0009	75.6655	67.0009	83.5002	0.7501
	DT	67.1111	67.225	67.1111	83.5556	0.6752
VGG-19	SVM	94.8889	94.9638	94.8889	97.4444	0.8010
	KNN	66.0007	75.0675	66.0007	83.0006	0.7304
	DT	67.2222	67.2651	67.2222	83.6111	0.6944
ResNet18	SVM	98.5556	98.5606	98.5556	99.2778	0.9677
	KNN	89.2222	89.3673	89.2222	94.6111	0.7383
	DT	68.4444	68.4734	68.4444	84.2222	0.6775

**Table 2:** Performance comparison of different models combined with SVM in defect detection of terahertz spectral.

Model	ML	Classes	Precision	Recall	F1_Score
AlexNet	SVM	Adhesion Failure	0.9574	0.9733	0.9653
		Layer Delamination	0.9932	0.9804	0.9866
		Void Defect	0.9532	0.9502	0.9516
VGG-16		Adhesion Failure	0.9401	0.9400	0.9401
		Layer Delamination	0.9771	0.9933	0.9851
		Void Defect	0.9356	0.9204	0.9277
VGG-19		Adhesion Failure	0.9460	0.9333	0.9396
		Layer Delamination	0.9863	0.9604	0.9730
		Void Defect	0.9167	0.9533	0.9346
ResNet18		Adhesion Failure	0.9932	0.9803	0.9866
		Layer Delamination	0.9868	0.9933	0.9900
		Void Defect	0.9768	0.9833	0.9801

## Data Availability

The original contributions presented in this study are included in the article. Further inquiries can be directed to the corresponding author.

## Abbreviations

The following abbreviations are used in this manuscript:

CWT	Continuous wavelet transform
SVM	Support vector machine
ResNet	Residual networks
Polymethacrylimide	PMI
PTFE	Polytetrafluoroethylene
THz-TDS	Terahertz time-domain spectroscopy
ReLU	Rectified Linear Units
VGG-16	Visual Geometry Group 16-layer network
VGG-19	Visual Geometry Group 19-layer network
KNN	K-Nearest Neighbors
DT	Decision Tree
